# Identification of an early transcriptomic signature of insulin resistance and related diseases in lymphomonocytes of healthy subjects

**DOI:** 10.1371/journal.pone.0182559

**Published:** 2017-08-04

**Authors:** Alice Matone, Eleonora Derlindati, Luca Marchetti, Valentina Spigoni, Alessandra Dei Cas, Barbara Montanini, Diego Ardigò, Ivana Zavaroni, Corrado Priami, Riccardo C. Bonadonna

**Affiliations:** 1 The Microsoft Research—University of Trento Centre for Computational and Systems Biology (COSBI), Rovereto, Italy; 2 Department of Medicine and Surgery, University of Parma, Parma, Italy; 3 Division of Endocrinology and Metabolic Diseases, Azienda Ospedaliero-Universitaria of Parma, Parma, Italy; 4 Department of Chemistry, Life Sciences and Environmental Sustainability, University of Parma, Parma, Italy; 5 Department of Mathematics, University of Trento, Trento, Italy; Virgen Macarena University Hospital, School of Medicine, University of Seville, SPAIN

## Abstract

Insulin resistance is considered to be a pathogenetic mechanism in several and diverse diseases (e.g. type 2 diabetes, atherosclerosis) often antedating them in apparently healthy subjects. The aim of this study is to investigate with a microarray based approach whether IR per se is characterized by a specific pattern of gene expression. For this purpose we analyzed the transcriptomic profile of peripheral blood mononuclear cells in two groups (10 subjects each) of healthy individuals, with extreme insulin resistance or sensitivity, matched for BMI, age and gender, selected within the MultiKnowledge Study cohort (n = 148). Data were analyzed with an ad-hoc rank-based classification method. 321 genes composed the gene set distinguishing the insulin resistant and sensitive groups, within which the “Adrenergic signaling in cardiomyocytes” KEGG pathway was significantly represented, suggesting a pattern of increased intracellular cAMP and Ca2+, and apoptosis in the IR group. The same pathway allowed to discriminate between insulin resistance and insulin sensitive subjects with BMI >25, supporting his role as a biomarker of IR. Moreover, ASCM pathway harbored biomarkers able to distinguish healthy and diseased subjects (from publicly available data sets) in IR-related diseases involving excitable cells: type 2 diabetes, chronic heart failure, and Alzheimer’s disease. The altered gene expression profile of the ASCM pathway is an early molecular signature of IR and could provide a common molecular pathogenetic platform for IR-related disorders, possibly representing an important aid in the efforts aiming at preventing, early detecting and optimally treating IR-related diseases.

## Introduction

Insulin resistance (IR), operationally defined as a reduction in insulin-mediated glucose metabolism [1], is commonly associated with obesity [2], ageing [3], male gender [4] and physical inactivity [5], and precedes and predicts Type 2 Diabetes Mellitus (T2DM) [6]. In the early stage, the euglycemic state is maintained by compensatory hyperinsulinemia [7], so that early alterations in insulin signaling at the cellular level may occur also in apparently healthy subjects [8]. The consequences of chronic IR/hyperinsulinemia are believed to be far reaching, and not limited to T2DM pathogenesis [6], but also to other conditions, including atherogenic dyslipidemia [9], hypertension [10] and atherosclerosis itself [11, 12]. This has been encapsulated in the concept of metabolic syndrome [13, 14], or cardiometabolic risk [15], a clinically recognizable high risk state for T2DM and cardiovascular disease, which are believed to be pathogenically rooted in the common soil of IR [14, 15]. The list of other conditions associated with IR is impressively long and diverse, and includes, without being limited to, Alzheimer’s disease (AD) [16] and chronic heart failure (CHF) [17, 18].

The early molecular alterations leading to IR in humans are known only in part. One well grounded hypothesis holds that increased diacylglycerol and activation of several protein kinase Cs are central to the development of IR in a number of conditions such as obesity, T2DM, non alcoholic fatty liver disease, and have been detected also in lean old individuals and lean first degree relatives of patients with T2DM [19]. However, this scenario might be unable to explain why, for instance, in some individuals, pancreatic beta cells fail to compensate IR and T2DM develops [20], *i*.*e*. it may need a second “hit” to account for the dire consequences of IR. Another hypothesis, supported by a wealth of convincing experimental evidences, puts FOXO proteins, in particular FOXO1, at the heart of the pathogenesis of both liver/hypothalamus IR and beta cell demise [21, 22]. However, FOXO1 emerges as a key transducer to most, but perhaps not all [23], detrimental consequences of IR, leaving unanswered the question of what causes IR in the first place. Of course the combination of the two hypotheses would be endowed with fully explanatory power, according to a “plural pathway” mechanism.

Both the above mentioned hypotheses are extensions of working hypotheses [24, 25] aiming at explaining findings in a single, specific organ known to be involved in the pathogenesis of IR and T2DM. However, in the case of heterogeneity/multiplicity of the mechanisms of IR, by focusing at the beginning on a single tissue there might be an inadvertent selection bias in favor of pathogenetic mechanisms preferentially and/or primarily at work in that tissue and against possible generalized, widespread mechanism(s).

Peripheral blood mononuclear cells (PBMCs) express approximately 80% of the genes encoded by the human genome [26]. Furthermore, the continuous contact of PBMCs with both endogenous and exogenous cues puts them in a unique position to recapitulate the overall genomic response of the individual to the environment. Thus, PBMC gene expression profile may be a useful, accessible window on the pathophysiology of processes occurring in hardly accessible organs and tissues [27]. A few studies of PBMC gene expression analysis have been performed to detect cross-sectionally the associations with dietary patterns in healthy individuals or to monitor the changes induced by dietary intervention in obese, insulin-resistant individuals [28–31].

We hypothesized that, if IR induces ubiquitous changes in gene expression patterns, circulating mononuclear cells may be reporters of this phenomenon. The aim of our study, therefore, was to assess whether “primary” (i.e. not associated to obesity and/or metabolic syndrome) IR is characterized by a specific pattern of gene expression profile in PBMCs. To reach this goal, we analyzed 40 healthy subjects in two different comparisons. Initially, we selected 20 lean healthy individuals from the extremes of the distribution of HOMA-IR (Homeostasis Model Assessment-estimated Insulin Resistance), a recognized and widely used index of IR [32], within a cohort of carefully phenotyped healthy subjects, after matching for BMI (Body Mass Index), age and sex. In these two polar groups, with a hugely different degree of insulin sensitivity, we explored, with a microarray based approach, the gene expression profile of PBMC by an innovative rank-based classification method [33, 34] and we discovered a biomarker of “primary” IR. After identifying a specific pathway as the putative originator of the biomarker of “primary” IR, we tested further whether alterations in gene expression of this pathway could be considered as a biomarker of IR. As a first step, we tested whether the ASCM pathway tracks with insulin resistance also in 20 otherwise healthy overweight/obese people. Secondly, we investigated whether the same pathway could represent a biomarker of organ damage related to IR, by testing its expression pattern in clinical IR-related disorders, such as T2DM [35], CHF [36, 37] and AD [38] (from publicly available data sets).

## Results

### Characteristics of the study population

The main features of the whole study cohort are reported in S1 Table. All subjects had normal glucose regulation, with fasting plasma glucose <100mg/dl and 2h plasma glucose after OGTT < 140mg/dl (ADA guidelines). The insulin-sensitive (IS) and the insulin-resistant (IR) groups were selected from the entire cohort as described in the Methods section. The main characteristics of the IS (n = 10) and of the IR (n = 10) groups are reported in Table 1. No statistically significant differences in the variables composing the metabolic syndrome/cardiometabolic risk (glucose, waist, blood pressure, triglycerides and HDL-cholesterol) were detected between the IS and the IR groups. The IR group, however, had a mean homeostatic model assessment (HOMA)-IR value 8-fold higher than the IS group (p<0.001), to highlight a huge difference in insulin sensitivity.

**Table 1:** Anagraphic, anthropometric and clinical characteristics of the low BMI insulin-sensitive (Low HOMA-IR) and insulin-resistant (High HOMA-IR) groups. N.s.: non-significant.

	Low HOMA-IR	High HOMA-IR	p-value
**Gender (M/F**) **(n)**	7/3	7/3	n.s.
**Age (years)**	40.2 ± 11.3	33.9 ± 6.1	n.s.
**BMI (Kg/m2)**	22.5 ± 2.2	22.2 ± 1.6	n.s.
**HOMA-IR (pure number)**	0.5 ± 0.1	4.0 ± 2.8	p<0.001
**Systolic blood pressure (mmHg)**	115.4± 11.9	111.7 ± 15.0	n.s.
**Diastolic blood pressure (mmHg)**	75.4±9.2	75.7± 8.2	n.s.
**Total Cholesterol (mg/dl)**	208.3±38.3	199.2±32.5	n.s.
**HDLc (mg/dl)**	65±11.2	57.7±16.9	n.s.
**LDLc (mg/dl)**	129.44±38.0	124.8±31.6	n.s.
**Triglycerides (mg/dl**)	69.3±45.8	83.5±54.5	n.s.
**hsPCR (mg/l)**	0.86±1.0	0.58±0.3	n.s.
**Waist (cm)**	84.0±7.0	84.4±8.5	n.s.

### Gene expression microarray analysis: identification of a biomarker of insulin sensitivity/resistance

**Fig 1:**
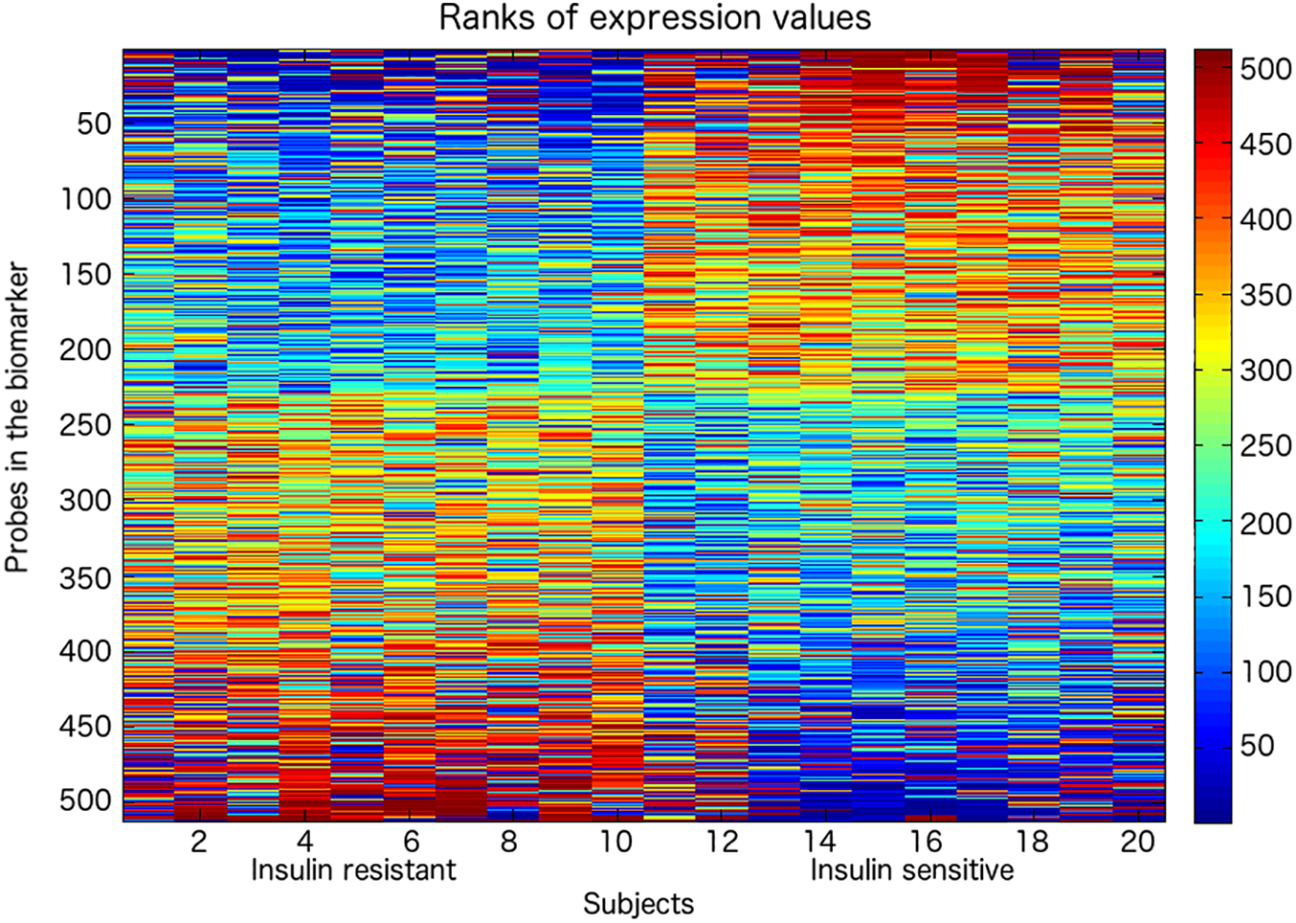
Heatmap of the 512 probes distinguishing IR and IS subjects. Rank values of the probes are represented and colors indicate the position of the probe expression level in the ranking of each sample (ascending order, red for over expressed probes, blue for under expressed probes). Each column reports the data of a single subject: 1 to 10 are the insulin resistant individuals, 11 to 20 are the insulin sensitive subjects. See also S2A Table.

**Fig 2:**
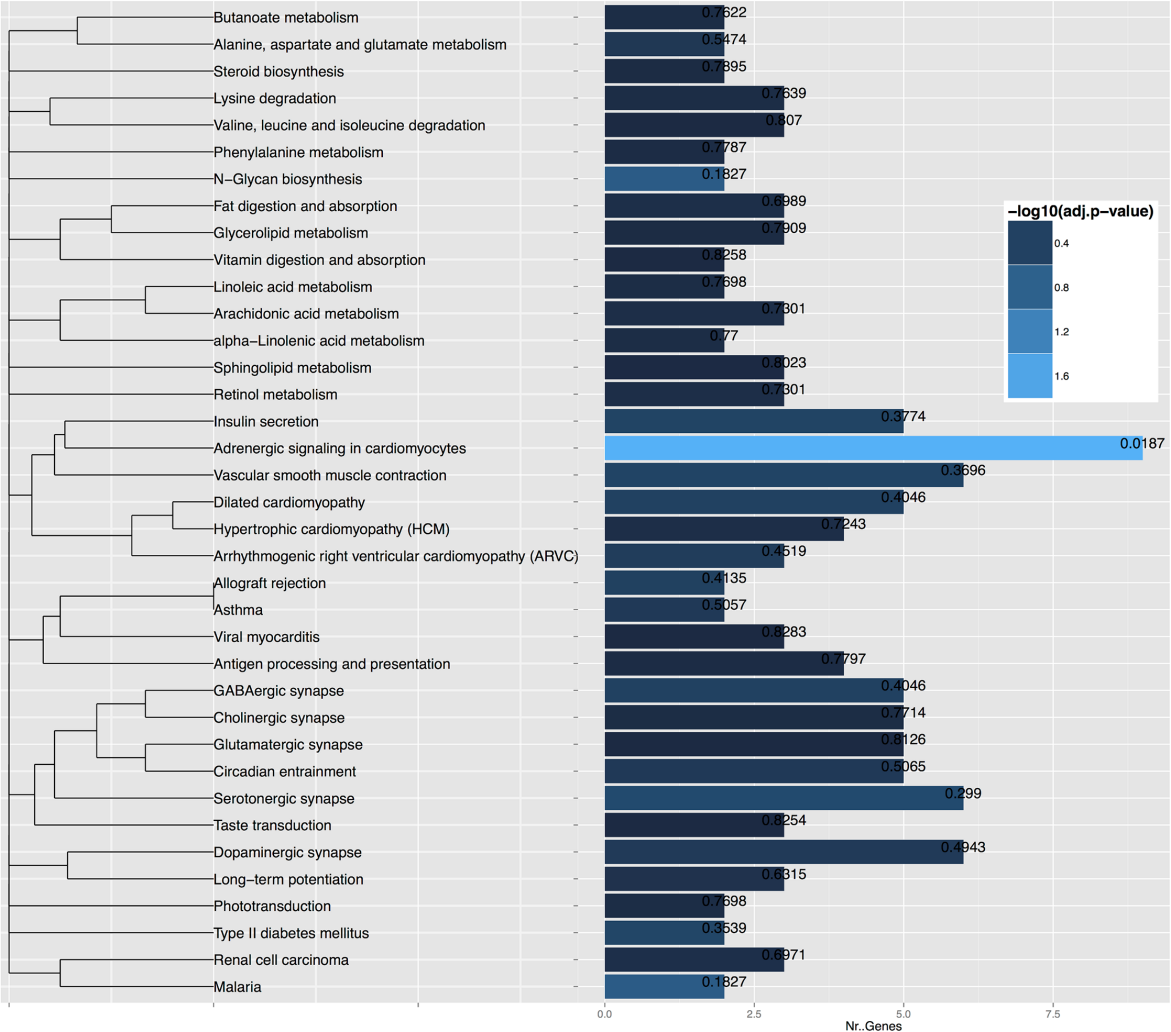
Bar chart of gene enrichment. The bars represent the annotated KEGG pathways within the genes distinguishing between IR and IS subjects. The Bonferroni corrected p-value is reported on each bar, while the bar color represents the –log10 (p-value) for each term. The bars’ length shows the number of genes found in each term. The dendrogram on the left represents the term similarity, *i*.*e*. terms are connected depending on the number of genes they share.

The biomarker computation was carried out by an enhanced version of the rank-based classification method introduced in [32, 33] as described in the Methods section. The IS and IR groups of subjects could be distinguished by a biomarker of 512 probes with an accuracy of 100%, according to a 5-fold cross-validation approach and a permutation test p-value of 0.005 (S2A Table). Fig 1 shows the heat map for the 512 probe expressions in the 20 subjects. Of these 512 probes, 321 could be mapped to genes (S2B Table reports the 321 genes and the associated fold change and p-value). KEGG (Kyoto Encyclopedia of Genes and Genomes) pathway enrichment analysis for the 321 genes showed significant enrichment (Bonferroni adjusted p-value 0.0187) of the Adrenergic Signaling in CardioMyocytes (ASCM) KEGG pathway [39–41], of which 9 genes (Table 2), with significantly altered expression in the comparison between the IS and the IR groups, are represented in the list of 321. Other 36 KEGG pathways were identified after functional analysis, among which insulin secretion and T2DM, but they did not have significant p-value after adjustment. Fig 2 shows the bar charts for the KEGG pathway annotation, representing the number of genes found in each pathway and the corrected p-value associated with them.

**Table 2:** Differentially expressed genes associated to the ASCM pathway included in the biomarker. The table provides the list of gene symbols, the log2 fold-change of gene expression levels between IS and IR subjects and the associated Wilcoxon-test p-values.

Gene symbol	Log2FC [IS/IR]	p-value	direction
**ADCY9**	-0.295	0.0499	Up regulated in IR
**TNNI3**	0.213	0.0499	Up regulated in IS
**RAPGEF3**	-0.085	0.0281	Up regulated in IR
**CACNA1S**	0.068	0.0104	Up regulated in IS
**CACNG3**	-0.090	0.0379	Up regulated in IR
**ADRA1B**	0.095	0.0499	Up regulated in IS
**CAMK2D**	-0.200	0.0499	Up regulated in IR
**PPP2R3C**	0.174	0.00466	Up regulated in IS
**PPP2CA**	-0.180	0.00466	Up regulated in IR

Increasing evidences indicate that in obese subjects mononuclear cells can induce chronic low-grade inflammation, which develops into insulin resistance and metabolic syndrome [42]. Then we asked whether the alteration of the ASCM pathway is detected also in overweight subjects with high HOMA-IR index. For this reason, from the MultiKnowledge cohort, we selected other two groups of 10 subjects with different values of HOMA-IR index but with BMI>25.0 (namely IS-BMI and IR-BMI), and matched for age, sex and BMI, as described in the methods section (Table 3). Then, we tested whether the expression of some of the genes in the ASCM pathway could provide a biomarker able to discriminate between the two high BMI groups.

**Table 3:** Anagraphic, anthropometric and clinical characteristics of the high (BMI>25.0) BMI insulin-sensitive (Low HOMA-IR) and insulin-resistant (High HOMA-IR) groups. N.s.: non-significant.

	IS-BMI	IR-BMI	p-value
**Gender (M/F) (n)**	6/4	7/3	n.s.
**Age (year)**	36.70± 4.7	38.10± 6.9	n.s.
**BMI (Kg/m2)**	27.7± 2.5	27.9 ± 2.7	n.s.
**HOMA-IR (pure number)**	0.8 ± 0.3	3.1 ± 0.8	p<0.001
**Systolic blood pressure (mmHg)**	124.0±11.0	122.6 ± 13.7	n.s.
**Diastolic blood pressure (mmHg)**	77.2±5.5	82.9± 7.0	n.s.
**Total Cholesterol (mg/dl)**	200.5±38.3	211.5±37.2	n.s.
**HDLc (mg/dl)**	52.2±8.0	47.3±12.3	n.s.
**LDLc (mg/dl)**	134.6±31.8	145.1±43.8	n.s.
**Triglycerides (mg/dl)**	68.4±30.2	119.0±48.7	p<0.05
**hsPCR (mg/l)**	1.35±0.99	3.0±4.2	n.s.
**Waist (cm)**	94.40±7.3	98.1±5.0	n.s.

**Fig 3:**
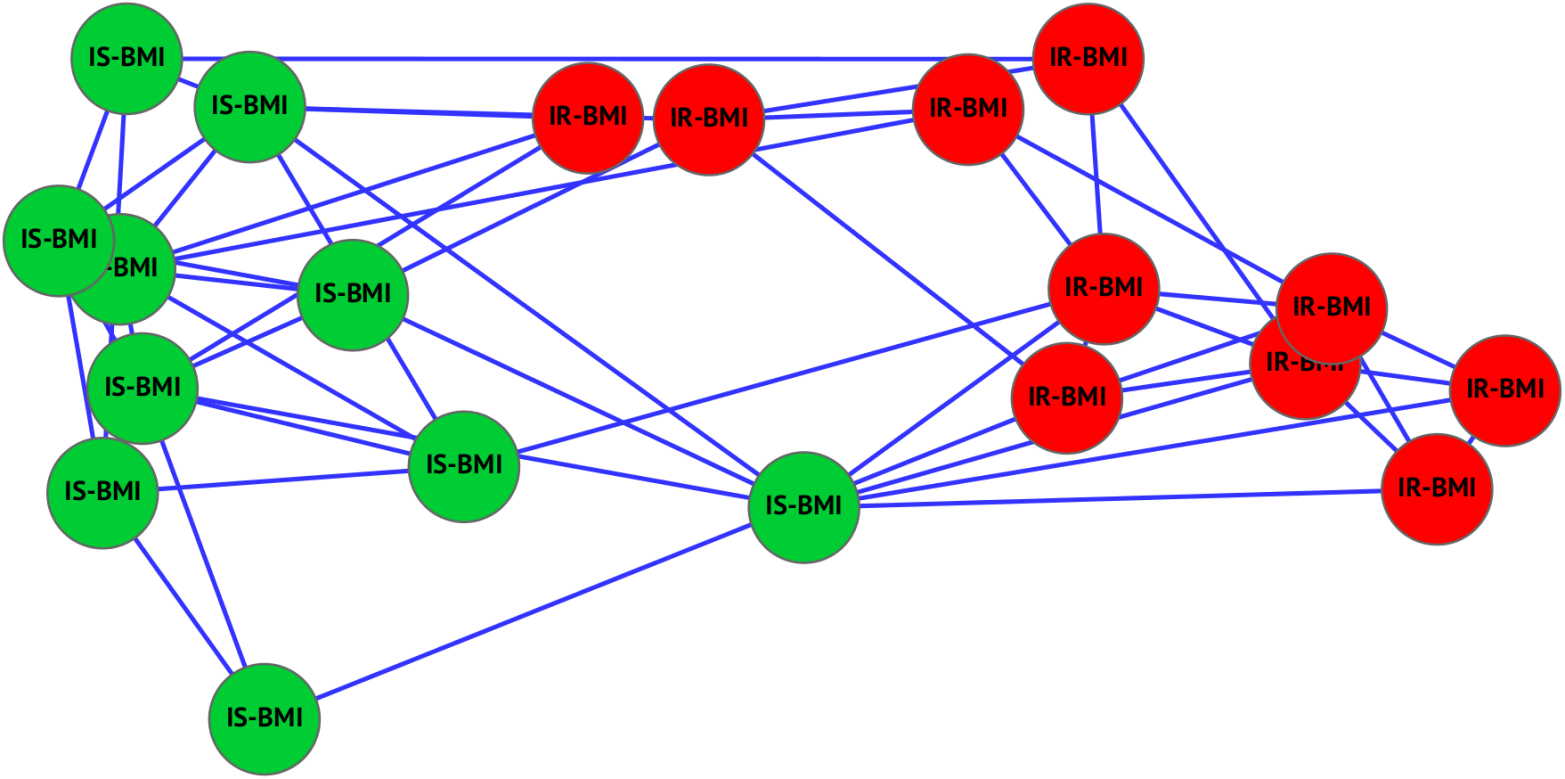
Network of subject groups in the IS-BMI and IR-BMI study of the ASCM pathway. Networks represent subjects’ classification in the validation study of the ASCM pathway in groups with high BMI (IS-BMI and IR-BMI). Nodes represent subjects (green ones are IS-BMI subjects, red ones are IR-BMI). The edge length is proportional to the difference between the subject signatures (short edges indicate high similarities, long edges indicate low similarities, no edge indicates a negligible similarity). In each case subjects naturally cluster together according to the class they belong to. See also S3 Table.

Of the 96 probes within the ASCM pathway, 16 probes, corresponding to 15 genes, (S3 Table), constituted a biomarker that distinguished IS-BMI group from IR-BMI group with 100% discriminant accuracy and a permutation test p-value of 0.0001 (Fig 3).

Deepening in the gene expression dysregulations of the ASCM pathway, our working hypothesis was the net functional balance of the differentially expressed genes in this pathway would predict a cluster of alterations in the IR group, including increased intracellular cAMP (due to overexpression of ADCY9) and Ca^2+^ (due to an alteration of the expression of PP2A subunits PPP2R3C and PPP2CA) levels and accelerated apoptosis (due to an overexpression of RAPGEF3). These alterations would seem to depict a scenario in which individuals with “primary” (i.e. not associated to obesity and/or metabolic syndrome) IR are burdened with “overwork” (increased intra-cellular cAMP and Ca^2+^) in excitable cells, together with accelerated cellular demise. Since IR is a risk factor for T2DM, CHF and AD, diseases in which excitable cells (beta cells, cardiomyocytes and neurons, respectively) play major roles, we hypothesized that, if an alteration of the gene expression pattern in the ASCM pathway is associated with the development of IR, within its genes we should find biomarkers able to distinguish healthy from diseased also in these conditions.

**Fig 4:**
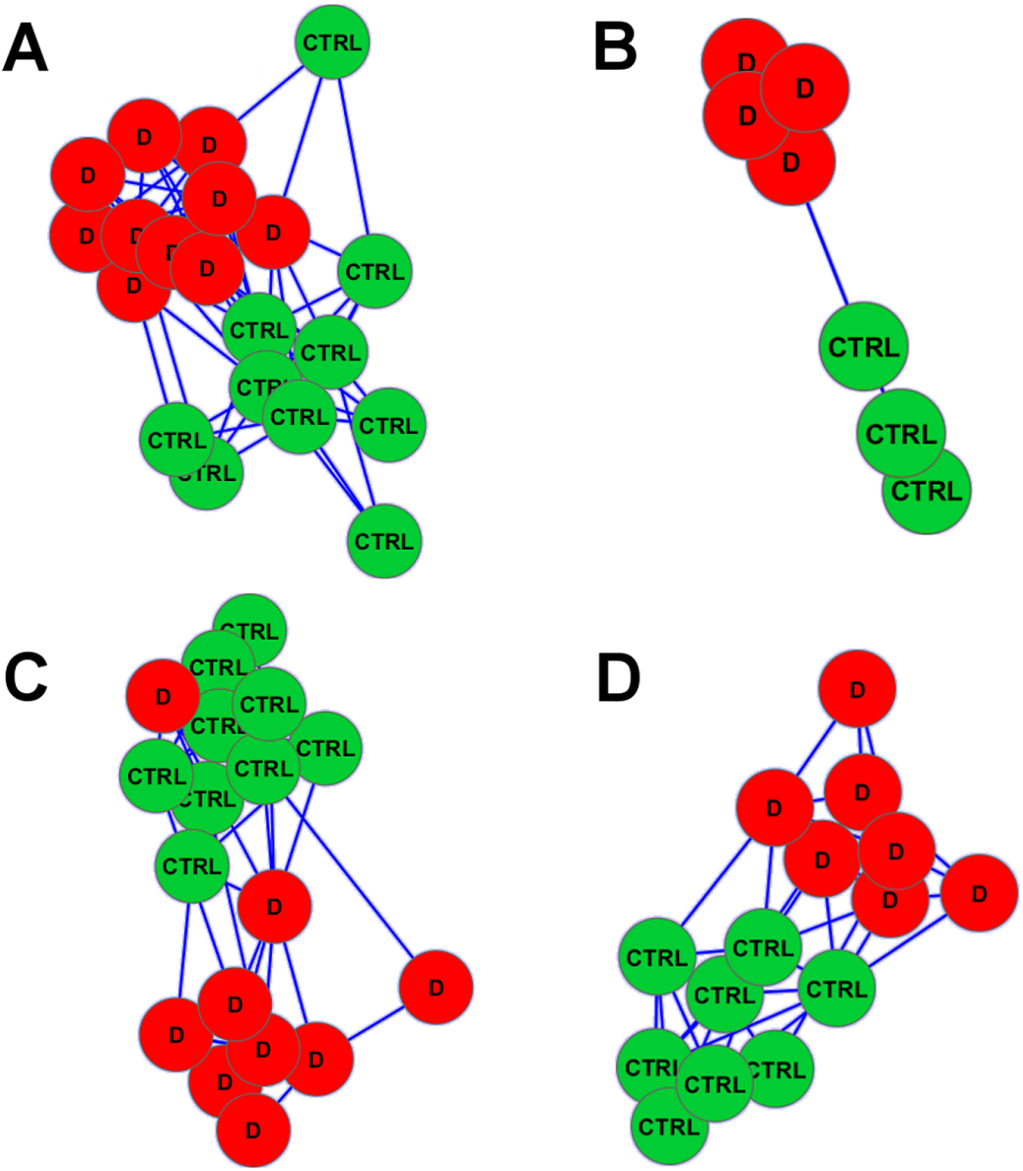
Networks of subject groups in each replication study of the ASCM pathway. Networks represent subjects’ classification in the four replication studies of the ASCM pathway as a provider of classification effective biomarkers of IR-related disorders. Nodes represent subjects (green ones are healthy subjects, red ones are diseased). The edge length is proportional to the difference between the subject signatures (short edges indicate high similarities, long edges indicate low similarities, no edge indicates a negligible similarity). In each case subjects naturally cluster together according to the class they belong to. A: classification of 10 healthy donors and 10 T2DM donors [35]. B: classification of 3 healthy controls versus 4 NICM patients [36]. C: classification of 9 healthy controls versus 9 CHF patients [37], in this case a diseased subject is miss-classified by the algorithm. D: classification of 8 control donors and 7 AD patients’ autopsies [38]. Networks were drawn through Cytoscape [40]. See also S4 Table.

To test this hypothesis, we investigated whether the genes in the ASCM pathway could be used to find a biomarker that would distinguish healthy controls from patients with T2DM, or CHF or AD. As to T2DM, we tested gene expression data of pancreatic beta cells of 10 healthy donors and 10 T2DM donors from a publicly available dataset (GSE20966 [35]). Of the 418 probes within the ASCM pathway, 52 probes, corresponding to 43 genes (S4A Table), constituted a biomarker that distinguished controls from patients with 100% discriminant accuracy according to a 10-fold cross-validation approach and a permutation test p-value of 0.0001 (Fig 4A).

As to CHF (of which IR is a known risk factor), we used two publicly available gene expression data sets (GSE9128 [36] and GSE21125 [37]) of PBMCs and white blood cells, respectively. In this case we used two datasets because the number of subjects of the first dataset was too small to allow the cross-validation of results. From [36] we compared 3 healthy controls versus 4 non-ischemic cardiomyopathy patients. In the gene expression data, 261 probes corresponded to genes in the ASCM pathway. Among these, 41 probes, corresponding to 37 genes (S4B Table), constituted a biomarker that distinguished healthy controls from patients with 100% discriminant accuracy and a permutation test p-value of 0.026 (Fig 4B). From [37] we compared 9 healthy controls versus 9 CHF patients. In the gene expression data 146 probes corresponded to genes in the ASCM pathway, within which 22 genes (S4C Table) constituted a biomarker that distinguished controls from patients with 91.7% discriminant accuracy according to a 9-fold cross-validation approach and a permutation test p-value of 0.0015 (Fig 4C).

As to AD, we tested gene expression data of hippocampal cells from a publicly available dataset (GSE28146, [38]) taken from 8 control donors and 7 AD patients’ autopsies. In this case, within the 261 probes of the ASCM pathway, 15 genes (S4D Table) constituted a biomarker that distinguished controls form patients with 100% discriminant accuracy according to a 5-fold cross-validation approach and a permutation test p-value of 0.0002 (Fig 4D).

**Fig 5:**
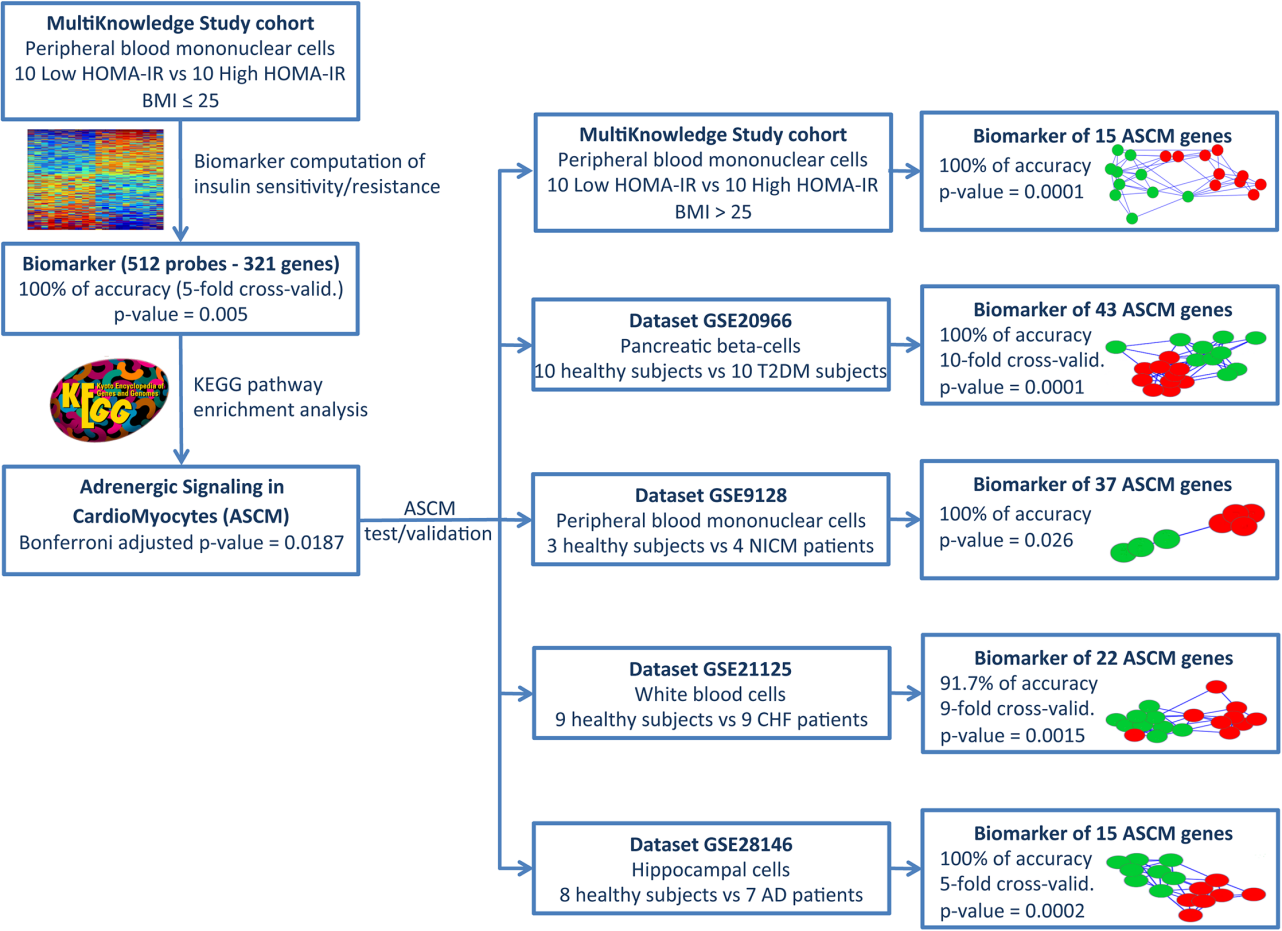
Block diagram reassuming the results of the paper. Starting from the MultiKnowledge population we identified a cohort of 10+10 subjects with normal BMI and opposite HOMA-IR value. We computed a biomarker able to discriminate between the two groups and we identified the ASCM pathway as the most significant pathway in the biomarker. We then validated this result in another cohort from the MultiKnowledge study of 10+10 subjects with BMI>25 and opposite HOMA-IR value. Finally, we identified genes in the ASCM able to discriminate between healthy controls and patients with T2DM, or CHF, or AD.

The key findings are summarized in Fig 5.

## Discussion

One main finding of this study is the identification of a PBMC transcriptomic signature exclusively associated to IR, independently of age, sex, BMI, waist, blood pressure and lipids. Fig 5 is a concept map, which highlights the principal findings of the paper. These results were obtained by means of an innovative ranked-based classification method. The transcriptomic signature of IR included 321 annotated genes, which identified IR and IS subjects with 100% accuracy. The most representative pathway, and the only one endowed with full statistical significance, was the "Adrenergic signaling in cardiomyocytes" (ASCM) KEGG pathway, therefore the best candidate for having a role within IR development. Since alterations in the ASCM pathway were observed also in overweight/obese subjects with polar opposite level of HOMA-IR index, gene expressions in this pathway should be directly related to insulin resistance and not a feature related to BMI. At a closer scrutiny, the high IR-BMI group down-regulates genes involved in the betaA2R signaling (GNAI1 and PI3KCG), decreasing the PI3K activity [43]. This observation is in line with the insulin sensitizing activity of PI3K and strongly suggests that the modification of the expression of genes related to this pathway could be a biomarker of IR. If this hypothesis holds true, the ASCM pathway should harbor biomarkers distinguishing between healthy from diseased subjects also in other cases where IR is known to develop.

Specifically, the differentially expressed genes of the ASCM pathway are involved in the modulation of the calcium flux across the membranes (CACNA1S, CACNG3, CAMK2D), and in cell growth and apoptosis (RAPGEF3, PPP2R3C, PPP2CA, TNNI3). After scrutinizing the alterations in the expression of genes involved in this pathway, we put forward the hypothesis that they should lead to an increase in intracellular cAMP and calcium and to accelerated apoptosis and impaired cell growth in the IR group. These processes are extremely relevant in disorders of excitable cells, among which IR is believed to play a significant role in T2DM (beta cells), CHF (cardiomyocytes) and AD (neurons).

Thus, we investigated whether the ASCM pathway gene expression profile could distinguish affected individuals from healthy controls, i.e. we tried to replicate the role of the pathway investigating whether it would contain biomarkers of IR-related disorders. For this purpose we applied a ranked-based classification method to validate our findings and found that the ASCM gene expression profile could provide molecular biomarkers of all the above mentioned conditions (Fig 5).

With hindsight, these results are not surprising. For instance, as to T2DM, it is well known that calcium plays a key role in the process of glucose-induced insulin release of the pancreatic beta cell [44]. Mice with chronic activation of CAMK2 in beta cells developed glucose intolerance and abnormal insulin secretion through a ryanodine receptor 2 (Ryr2) dependent mechanism. Chronic activation of Ryr2 was also implicated in beta cell apoptosis [45]. In the T2DM biomarker herein reported, CALML3, CALML5 and CAMK2B genes are up regulated in the patients, possibly leading to an aberrant activation of RyRs. Studies in diabetic rat and human samples have shown that RyRs activity can be regulated during acute hyperglycemia in cardiomyocytes [46]. Moreover, it was demonstrated that sarco/endoplasmic reticulum Ca^2+^-ATPase (SERCA) activity and calcium content are reduced in myocytes of a mouse model of diabetes (db/db mice), and similarly in glucose intolerant rats [47, 48].

Also in the case of CHF, a biomarker derived from the ASCM pathway was able to discriminate between patients and controls, in both CHF gene expression data sets. According to our results in IR vs IS, the adrenergic receptor signaling via cAMP-dependent and independent pathways may be overactive and it is known that high intracellular calcium levels, one of the main features of CHF, result into increased apoptosis. In different rat models of IR, the decreased SERCA activity is caused by a slowed cytosolic Ca2+ removal [49], which leads to alterations of the excitation–contraction coupling process. Finally, IR is present in non-diabetic CHF patients with both reduced and preserved ejection fraction [50].

Also in the AD case a number of differentially expressed genes of the ASCM pathway could distinguish patients from controls. Aberrant intracellular calcium signaling occurs in the early stage of AD, and elevated cytosolic calcium concentrations correlate with amyloid precursor protein production [51]. Recent studies showed that IR and the associated key risk factors (such as obesity and physical inactivity) are associated with both brain changes and an increased risk of developing dementia or AD [52]. Moreover, in mouse models of AD the expression of RyRs is up-regulated in neurons [53].

Thus, these replication studies confirm that the ASCM pathway gene expression profile provides biomarkers of IR and IR-related conditions and that ASCM plays a role in the pathogenic processes put in motion by “primary” IR. To this regard, it is to be highlighted that in our study the ASCM pathway was selected in otherwise healthy subjects, with normal BMI and clinical parameters, and in cells (PBMC) that are not classical targets of insulin action, are exposed to all cues circulating in the blood stream and express about 80% of the overall genome. Thus, our findings strongly suggest a very early and widespread involvement of the ASCM pathway in the pathogenesis of IR and related conditions. The putative functional alterations brought about by the IR related changes in the ASCM pathway can be predicted to be particularly relevant in excitable cells, such as pancreatic beta cells, myocytes and neurons. The ASCM pathway, therefore, could be (one of) the molecular basis of vulnerability of excitable cells secondary to, or concomitant with, the onset of IR.

It could be argued that within the list of genes distinguishing IR and IS subjects, the canonical candidates that are usually altered in IR are missing, such as some inflammatory genes, insulin receptors or glucose channels. On one side we can say that there are a few genes within the list that are indeed ascribable to IR, such as RIMS2 which has a role in insulin secretion [54], SLC2A2 (GLUT2, a glucose channel [55], up-regulated in IS), IRS4 (insulin receptor substrate 4 [56], up-regulated in IR) and IL4, (up-regulated in IS). On the other hand, it must be kept in mind that the analyzed subjects are healthy and young individuals, it is therefore not surprising that a massive alteration is not detected in the gene expression profile, but rather a somewhat softened signal which is to be attributed to mechanisms generally diffused in the organism and early in the history of IR development.

It could be argued that a comparison between two groups of 10 subjects each cannot reach the proper statistical power. However, this is due to the fact that we made a very closed match between the subjects in each group in order to avoid confounding effects of the principal features involved in the onset of IR. This type of approach is consistent with other previous papers [57, 58]. To overcome the problem, when possible, classification accuracy of computed biomarkers has been evaluated according to a state-of-the-art cross-validation scheme and by computing permutation test p-values that in all cases confirmed the statistical significance of the analysis (see Results and Methods sections). Moreover, we also confirmed our results in another well matched population of 20 subjects, thus increasing the effectiveness of the overall analysis, which finally considers 40 subjects in total.

One main limitation of this study is the lack of a post-hypothesis specific experimental investigation. Although our results derive from solid microarray data, the connection to specific protein activity cannot be abstracted, for instance because of post-translational modification. Therefore the herein generated hypothesis would need further support from targeted proteomics examination in healthy and diseased states.

We conclude that modifications in the gene expression profile of the ASCM pathway are a very early molecular signature of “primary” IR and that the gene expression profile of this same pathway provides biomarkers of several IR-related conditions. Corollaries of the findings herein reported are that the ASCM pathway could provide a common molecular pathogenetic platform for IR-related disorders as diverse as T2DM, CHF and AD and that the early molecular signature of IR could be an important aid in the efforts aiming at preventing, early detecting and optimally treating IR-related diseases.

## Methods

### Study subjects

The study population included 148 apparently healthy volunteers selected among the offspring of the Barilla study population [12, 59]. The Barilla study is a longitudinal observational survey performed in 732 factory workers of the Barilla factory since 1981 with a follow up of 15 years. In 2007–2008 we launched another observational study with the preferential recruitment of the offspring of the participants of the Barilla Study, naming it Multiknowledge study. The general aim of this study was to select and to investigate novel potential biomarkers of the cardiometabolic risk, with specific attention devoted to the phenotype of hyperinsulinemia/insulin resistance. Exclusion and inclusion criteria of the Multiknowledge study were extensively described in previous reports [60, 61]. Briefly, Caucasian subjects from both genders between 18 and 60 years of age were enrolled. They underwent medical history collection and physical examination, including smoking habits, blood pressure and anthropometric measurements. After an overnight fast, a venous blood sample was drawn for complete blood cell count, standard clinical chemistry and other measurements for companion experiments, including 50 ml of blood in an EDTA BD Vacutainer tube (Becton Dickinson and Company, Franklin Lakes, NJ, US) for isolation of circulating PBMC (see below for further details). Subsequently, a standard oral glucose tolerance test (OGTT) (75 g of glucose administered orally) was performed in all subjects and plasma glucose and serum insulin levels were measured to assess glucose tolerance. Complete data for this report were available in 148 subjects.

This study was reviewed and approved by the Institutional Ethical Committee "Comitato Etico per Parma". All subjects were informed about the aims and the potential risks of the study and signed a written, informed consent. The main demographic, anthropometric and humoral features of the study cohort are reported in S1 Table.

Since the Multiknowledge Study is a purely observational survey with a non-trial design, according to the policy of the International Committee of Medical Journal Editors (ICMJE), registration of the Multiknowledge Study in a public trials registry is not required (http://www.icmje.org/recommendations/browse/publishing-and-editorial-issues/clinical-trial-registration.html).

### Assessment of insulin sensitivity/resistance and selection of insulin-sensitive and insulin-resistant groups

HOMA-IR was calculated using the formula:

$$HOMAIR=\frac{fasting\;glucose*fasting\;insulin}{22.5}$$ (1)

in which glucose units are mmol/l and insulin units are mU/l.

After excluding all subjects with BMI≥25.0, we selected 10 subjects from the bottom range (IS group) and 10 subjects from the top range (IR group) of the HOMA-IR distribution, with the additional goal of matching the two groups also for anthropometry, sex, blood pressure, triglycerides and HDL-cholesterol, i.e. the phenotypes of the metabolic syndrome. Selected features of the IS and IR groups are reported in Table 1.

A second selection was made in order to validate the results found in the first two groups (IS and IR). We selected from the same cohort all subjects with BMI>25.0 and, similarly to the first selection, we choose 10 subjects with the lowest HOMA-IR index (IS-BMI) and 10 subjects with the highest HOMA-IR index (IR-BMI). Both groups are matched for the principal anthropometry and clinical variables. Selected features of the IS-BMI and IR-BMI groups are reported in Table 3.

### Gene expression profiling of circulating lympho-monocytes

The PBMC transcriptome profile was assessed in IR and IS groups. PBMCs were immediately isolated by density gradient centrifugation (Lymphoprep, Sentinel Diagnostic, Milan, Italy), washed three times with PBS 1% FBS and then stored as a dry pellet at—80°C. Total RNA was isolated using column separation (RNeasy Mini Kit, Qiagen, Valencia, CA, US), followed by DNase treatment to avoid DNA contamination, according to the manufacturer’s instructions. RNA was then eluted with RNase-free water and stored at -80°C until hybridization.

Quality control testing of extracted RNA was performed before hybridization. Total RNA yield was checked using a NanoDrop ND-1000 UV-Vis Spectrophotometer (NanoDrop Technologies, Wilmington, DE, USA). RNA quality was assessed measuring the 28S/18S rRNA and the RNA Integrity Number (RIN) with a Bioanalyzer 2100, using RNA 6000 Nano Chips (Agilent Technologies, Santa Clara, CA, USA).

The gene expression microarray protocol was as previously described [62]. Briefly, a total of 500 ng of each RNA sample was reversely transcribed into cDNA and then amplified and labelled with Cy5 dye. The same amount of RNA from a commercially-available pool of human leucocyte total RNA (Clontech, Mountain View, CA, USA) was used as reference. Reference and sample RNA were hybridized together on 4X44 Whole Human Genome Agilent Microarray slides (Agilent Technologies, ref. G4112F). Dye-normalized, background-subtracted log-ratios of sample to reference expression were calculated using Agilent’s Feature Extraction Software v8.0. Hybridization quality was checked using the software’s quality report. We validated transcriptomic analysis by these methods in a previous paper [62].

### Statistical and bioinformatics analyses

All descriptive statistics are reported as mean ± SD, unless otherwise specified. The distributions of anthropometric and humoral parameters were tested by the Kolmogorov-Smirnov test to detect significant deviations from Gaussian distribution. If needed, log-transformation was applied to reduce/correct non-Gaussian distributions. Between-group comparisons were performed on normal variables using Student’s t-test. Statistical analysis for these clinical parameters was performed using software package SPSS 15.0 for Windows (SPSS Inc., Chicago, IL, US), except for the gene expression data which were read and cleaned by means of the R Bioconductor package Limma [63–65]. The functions readTargets and read.maimages were used to read the files. The backgroundCorrect function was used for background correction using the method “normexp”. Normalization within arrays and between arrays was performed with the functions normalizeWithinArrays (method “loess”) and normalizeBetweenArrays (method “quant”). The function avereps was used to average replicate probes, bringing the expression data set from 45015 to 41001 probes. Probe to gene mapping was achieved through Agilent annotation supporting files, BioMart [66], Gene cards [67], Aceview [68] and BLAST [69]. Data can be accessed at GEO (Gene Expression Ominbus) under the accession number GSE87005.

Biomarkers were identified by means of an enhanced version of the rank-based classification method proposed in [33, 34]. The method, extended with a global optimizer to compute the signatures providing the best sample classification according to genetic optimization, has been already successfully used [70, 71] and demonstrated to be a very efficacious tool during the SBV IMPROVER Diagnostic Signature Challenge, an international competition designed to assess and verify computational approaches for classifying clinical samples based on gene expression [72]. The method summarizes the characteristics of each sample through a rank-based signature of probes and then performs an all-to-all signature comparison by means of a distance metric based on weighted enrichment score (ES) [73, 74]. The results of the comparison are then collected in a distance matrix, which is used to classify samples into different groups by assigning each sample to the class whose elements have the lowest averaged distance. Moreover, to assess the reliability of the analysis, classification accuracy is computed, when possible, according to a cross-validation approach and its statistical significance is evaluated by a permutation test comparing the obtained classification accuracy with the accuracy of an empirical distribution obtained by 10000 random permutations of the probe labels. Finally, a biomarker is extracted considering the union of the probes included in at least one sample signature.

Cytoscape and the ClueGO plug in were used [40, 75] for gene annotation/gene enrichment analysis of KEGG pathways [41]. A Bonferroni step-down corrected p-value threshold of 0.05 was employed to declare the statistical significance of gene enrichment.

The above procedure was applied in a “reverse” fashion in the replication studies, the goal of which was to assess whether the ASCM pathway could provide statistically significant, classification effective biomarkers also in cells/organs/tissues taken from individuals with insulin-resistant conditions. After selecting all genes of the ASCM pathway, the algorithm was applied to test whether the pathway was significantly perturbed, more specifically whether some of its genes could form a biomarker, which would correctly classify patients and controls. For the second selection (BMI>25.0) and for each of the 4 datasets available in the scientific literature, a classification effective biomarker was found entirely made of genes belonging to the ASCM pathway. The statistical significance of the performance of these biomarkers was assessed by permutation tests (S3 and S4 Tables).

Cytoscape [40] was also used to draw the networks reported in Figs 3 and 4. In particular, the Edge-Weighted Force-Directed network layout (BioLayout) was used to arrange the placement of nodes according to subject distances. The R package ggplot2 was used for Fig 2.

## Supporting information

S1 Table General characteristics of the study cohort. The table represents the main characteristics of the subjects of Multi-Knowledge study. Data are reported as mean±standard deviation.
(PDF)

S2 Table **A, B. Features of the biomarker with statistically significant separation property between insulin-resistant and insulin-sensitive healthy individuals**. S2A Table: for each one of the 512 probes included in the biomarker, the probe ID, the associated gene symbol (when available, see S1B Table), the log2 fold-change of the gene expression level between IS and IR individuals and the associated Wilcoxon-test p-value are reported; S2B Table: the 321 biomarker features out of 512 for which a probe ID to gene symbol mapping is available. The list provides probe IDs, gene symbols, log2 fold-change of gene expression levels and associated Wilcoxon-test p-values between IS and IR.
(XLSX)

S3 Table Validation biomarker extracted from the ASCM pathway that can discriminate between IS-BMI and IR-BMI groups. The list provides probe IDs, gene symbols, log2 fold-change of gene expression levels between IS-BMI and IR-BMI individuals and their associated Wilcoxon-test p-values.
(XLSX)

S4 Table **A, B, C, D. Validation biomarkers extracted from the ASCM pathway that can discriminate healthy individuals from patients with IR-related conditions.** S4A Table: list of probe IDs, gene symbols, log2 fold-change of gene expression levels and associated t-test p-values in discriminating beta cells of patients with type 2 diabetes from healthy non diabetic individuals; S4B Table: list of probe IDs, gene symbols, log2 fold-change of gene expression levels and associated t-test p-values in discriminating PBMC of patients with non-ischemic cardiomyopathy from healthy non diabetic individuals; S4C Table: list of probe IDs, gene symbols, log2 fold-change of gene expression levels and associated t-test p-values in discriminating white blood cells of patients with chronic heart failure from healthy non diabetic individuals; S4D Table: list of probe IDs, gene symbols, log2 fold-change of gene expression levels and associated t-test p-values in discriminating hippocampal cells of patients with Alzheimer disease from healthy non diabetic individuals.
(XLSX)
