# Correction: Identification of an early transcriptomic signature of insulin resistance and related diseases in lymphomonocytes of healthy subjects

**DOI:** 10.1371/journal.pone.0211394

**Published:** 2019-01-23

**Authors:** Alice Matone, Eleonora Derlindati, Luca Marchetti, Valentina Spigoni, Alessandra Dei Cas, Barbara Montanini, Diego Ardigò, Ivana Zavaroni, Corrado Priami, Riccardo C. Bonadonna

There is an error in the Methods section under the sub-heading “Study subjects.” The first sentence of the second paragraph should read: This study was reviewed and approved by the Institutional Ethical Committee “Comitato di Etica dell’Università degli Studi di Parma.”
