# Supplementary material for: Identification of an early transcriptomic signature of insulin resistance and related diseases in lymphomonocytes of healthy subjects
Source: PLoS One. 2017 Aug 4;12(8):e0182559. doi: 10.1371/journal.pone.0182559 (PMC5544197; doi:10.1371/journal.pone.0182559)
Supplement: S1 Table — The table represents the main characteristics of the subjects of Multi-Knowledge study. Data are reported as mean±standard deviation. (PDF) [file pone.0182559.s001.pdf]

S1 Table. General characteristics of the study cohort.

Demographic, anthropometric and selected humoral features of the study cohort [n=148]	Mean ±SD
Gender [M/F]	82/66
Age [year]	37.0±18.0
BMI [Kg/m^2]	24.0±4.7
HOMA-IR [pure number]	0.69 ±0.2
Systolic blood pressure [mmHg]	130.0±14.1
Diastolic blood pressure [mmHg]	81.0±1.4
Total Cholesterol [mg/dl]	209.0±55.2
HDLc [mg/dl]	55.0±2.9
LDLc [mg/dl]	143.1.8± 55.9
Tryglicerides [mg/dl]	54.5±17.7
hsPCR [mg/l]	1.8±2.0

The table represents the main characteristics of the subjects of Multi-Knowledge study. Data are reported as mean±standard deviation.
